# Multispectral Filter Arrays: Recent Advances and Practical Implementation

**DOI:** 10.3390/s141121626

**Published:** 2014-11-17

**Authors:** Pierre-Jean Lapray, Xingbo Wang, Jean-Baptiste Thomas, Pierre Gouton

**Affiliations:** 1 LE2I Laboratory, University of Burgundy, Dijon 21000, France; E-Mails: pierre-jean.lapray@u-bourgogne.fr (P.-J.L.); jesse.xingbo@gmail.com (X.W.); pgouton@u-bourgogne.fr (P.G.); 2 Norwegian Colour and Visual Computing Laboratory, Gjøvik University College, Gjøvik 2818, Norway

**Keywords:** multispectral and color filter arrays, single solid state sensor, snapshot multispectral imaging, spatio-spectral scene sampling

## Abstract

Thanks to some technical progress in interferencefilter design based on different technologies, we can finally successfully implement the concept of multispectral filter array-based sensors. This article provides the relevant state-of-the-art for multispectral imaging systems and presents the characteristics of the elements of our multispectral sensor as a case study. The spectral characteristics are based on two different spatial arrangements that distribute eight different bandpass filters in the visible and near-infrared area of the spectrum. We demonstrate that the system is viable and evaluate its performance through sensor spectral simulation.

## 1. Introduction

According to the Oxford Dictionary of English [1], a spectrometer is an apparatus used for recording and measuring spectra, especially as a method of analysis. Humans' interest in spectroscopy dates far back, and we can notice in 1869 the use of a spectrohelioscope to view the solar corona by the astronomer P.J.C. Janssen [2]. However, the use of spectral imaging was not wide until the launch of the Landsat program by NASA in 1970s [3]. Since then, spectral imaging has found its place in scientific research, as well as in industrial and professional applications.

So far, man-made image sensors have not been able to acquire spectral information of incident radiation, but only an estimate of spatially-sampled and spectrally-weighted intensity values. Common linear and area image sensors are designed to sample a one- or two-dimensional optical image and convert it to an electronic image by means of optoelectronic conversion. As result, a scanning process, namely a series of exposures in the spectral and/or spatial domain, is very often necessary to acquire multiple images with different spectral domains.

This paper focuses on multispectral imaging (MSI—Table 1 shows all the accronyms in the paper) that acquires a few channels over broader bands that carry useful information in it and may cover visible, as well as invisible portions of the spectrum. This work presents an extended version of a preliminary characterization work that we presented at the Color and Imaging Conference in 2014 [4]. According to the literature [5], a multispectral image can be defined by an array of *X* rows, *Y* columns and *P* spectral channels. The multispectral values can be represented by several spectral components (*c_p_*) at each spatial location (*x*, *y*). The image is such that it would have been acquired through multiple sensors with different spectral sensitivities. We can define the system by:

(1) $$c_{p}\left(x,y\right)=\int_{\lambda }I\left(x,y,\lambda \right)\Phi_{p}\left(\lambda \right)d\lambda$$

where *p* indexes over channels, *λ* is the wavelength, *I*(*x*, *y*, *λ*) is the spectrally-dependent irradiance at each location and Φ*_p_*(*λ*) is the spectral sensitivity function for a given sensor response.

This approach is distinguishable from hyperspectral imaging, which produces narrow and contiguously-sampled spectra, aiming at an accurate radiance estimate, and is usually intended for different kinds of applications.

**Figure 1.**
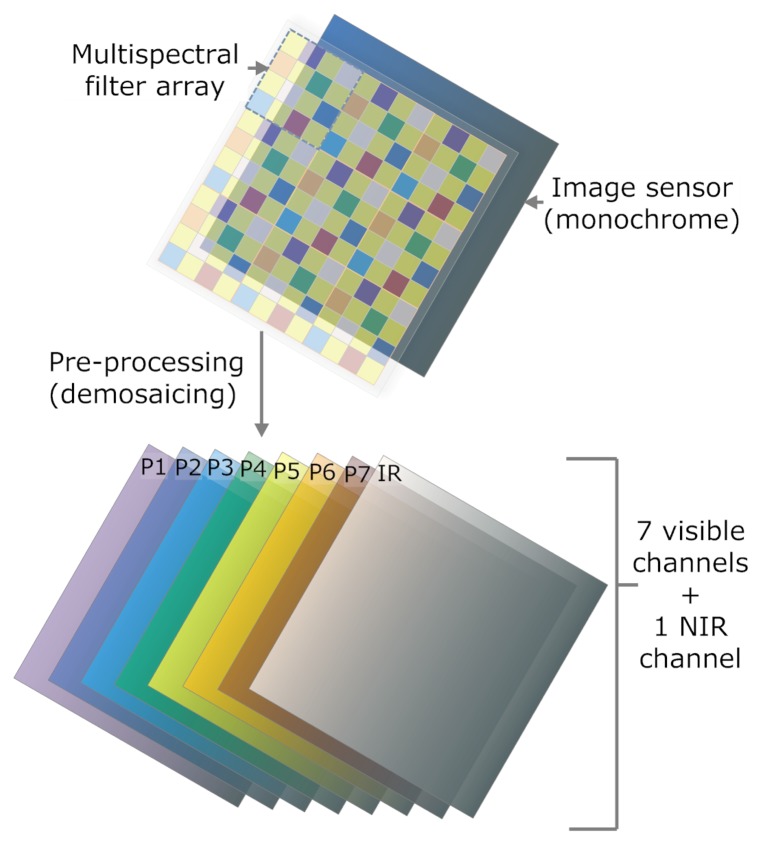
Global scheme of the multispectral imaging system. With the filter array technique, the filter is mounted on a common CMOS image sensor.

The scanning operation, *i.e.*, multiple exposures, often yields motion artifacts. In comparison, a snapshot imaging solution that captures a multispectral image at one exposure, *i.e.*, during a single sensor integration period, may avoid such artifacts. Although blur is by no means avoidable, it is easier to correct blur than to deal with artifacts due to multiple exposures [6], such as image registration. Further, non-scanning techniques do not rely on moving parts and, thereby, in many cases, lead to a simpler design, lower cost, higher portability and higher accuracy. For capturing multiband images with one exposure, the industry has been using the so-called color filter array (CFA) technique in digital color cameras. The Bayer arrangement [7] of the array is the most famous example. Similarly, multispectral filter array (MSFA) is the technique for capturing multispectral images where *p* > 3. An MSFA might be defined by its moxel, mosaic element, which corresponds to the occurrence of a pre-defined pattern that consist of a set of filters arranged geometrically in a relative manner. An overview of the global approach is shown in Figure 1.

In comparison with other snapshot MSI approaches, MSFA permits a simple and compact system. Although the mass production of such filter arrays remains to be seen, the concept of MSFA-based imaging solutions has particularly aroused the interest of academia and industry in designing and realizing such a sensor.

The purpose of this paper is to present the state-of-the-art of MSI/MSFA and to introduce as an example our new multispectral acquisition system, which consists of a standard sensor covered by a filter matrix. This matrix is typically able to let pass specific wavelengths from visible to near-infrared light.

The remainder of this paper is organized as follows. We begin in Section 2 by analyzing the state-of-the-art of MSI and MSFA sensors. In Section 3, we provide a comprehensive description of our solution through a sensor definition, a filter characterization and a spatial arrangement analysis of our new hardware. We also present our dedicated control tools that communicate with the sensor. In Section 4, we evaluate our solution and discuss the design constraints. Conclusions are provided in Section 5.

## 2. State-of-the-Art

**Figure 2.**
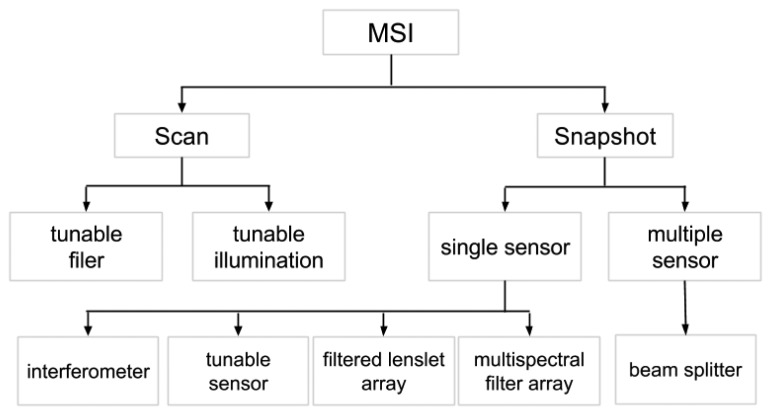
Classification tree of the main methods for multispectral capturing.

This section presents and discusses the different multispectral acquisition systems in the first part. Then, it focuses specifically on MSFAs. First, a classification of all of these systems is presented in Figure 2. We distinguish MSI systems by their ability to produce snapshot images, to use a single sensor or not and by the technology used to split the light.

### 2.1. Multispectral Imaging Systems

#### 2.1.1. Tunable Filters

Perhaps the most intuitive and well-studied examples of multispectral scanning techniques are the so-called tunable filters. By capturing an image of one spectral band at a time, a complete multispectral image is produced after a sequence of exposures.

**Figure 3.**
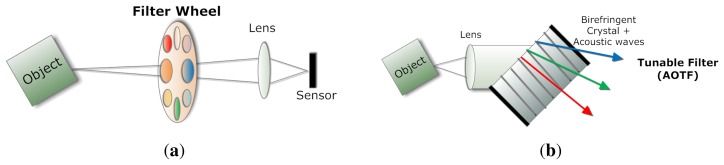
Schemes of the principles used by the techniques of filter wheel and liquid crystal tunable filters (LCTF). (**a**) Filter wheel with optical bandpass filters. Multispectral imaging is recorded by shifting the filters sequentially into the optical path; (**b**) Acousto-optical tunable filter principle. The acousto-optic effect is used to diffract and shift the frequency of light using sound waves.

A common and illustrative instance is the filter wheel (see Figure 3a), where a series of desired optical filters are installed [8]. When integrated with a monochromatic camera and typically inserted in the optical path, such a filter wheel may work in a synchronized way with the camera, so that one exposure corresponds to a certain type of filter, which yields a given spectral region or band.

The rotatory speed of such filter turrets is limited by their mechanical nature, therefore tunable filters whose spectral properties can be controlled electronically are developed [9]. Among various types of electronically-tunable filters, the two most used ones are liquid crystal tunable filters (LCTF) and acousto-optical tunable filters (AOTF). In fact, both of them provide notch passbands, but they differ in their principles [5]. The LCTF incorporates liquid crystal wave plate retarders tuned by applying electronic voltage within a Lyot birefringent filter, whereas AOTFs are solid-state birefringent crystals that vary in their response to an applied acoustic field [10] (see Figure 3b). The transition of these filters is much faster in comparison with the filter wheel solution. As an example, a multispectral imaging spectrometer with millisecond resolution has been developed based on the use of an AOTF and a progressive scan camera capable of snapshot operation for recording [11]. Color wheel and LCTF are also the common devices used in the multispectral imaging of cultural heritage objects in museums [12].

#### 2.1.2. Tunable Illumination

Another instantiation of a similar principle is to tune the illumination. Indeed, the spectral filters can be inserted anywhere in the optical path, such as at the illumination end. One of the main advantage of tunable illumination over the tunable filtering mentioned above is in the amount of energy arriving at the sample to measure, at the expense of a very rigid setup. This set up is widely used in cultural heritage object [13] and medical imaging [14], where material may be very fragile with respect to light radiation. To change the spectrum of the illumination, however, the physical filtering of a common light source is not the only solution. One may consider using different exposures under different light sources. Specifically, LEDs have been demonstrated to provide a convenient solution. Bouchard *et al.* present an LED-based system capable of high-resolution multispectral imaging at frame rates exceeding 220 Hz [15].

#### 2.1.3. Beam Splitting Techniques

**Figure 4.**
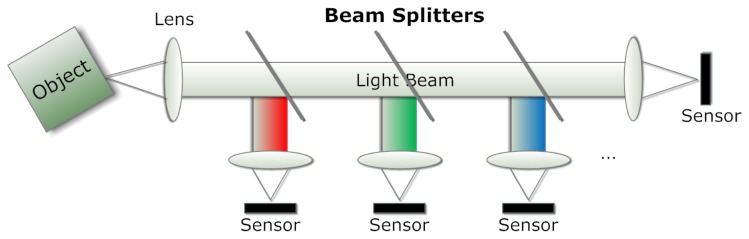
The design of a multi-spectral system. Three beam splitters with different wavelength ranges and three highly reflective mirrors.

The use of beam splitters in television cameras dates back to the 1950s when RCA introduced its massive three-tube color camera consisting of a configuration of three dichroic beam splitters that direct the incoming light into red, green and blue beams, each of which is projected through a lens onto a camera tube individually ([16], p. 45). A diagram of the principle is shown in Figure 4. To reduce the complexity and the dimensions of a color camera, Lang and Bouwhuis proposed a prism assembly for Plumbicon camera in 1965 [17]. This camera comprises three prism blocks and makes use of total internal reflection and dichroic filters.

Intuitively, the technique of beam splitting may generate more than three beams. A general multispectral beam splitting method is introduced in [18] where a three-band example is presented. Later, a similar approach was developed that employs a stack of dichroic filters, thus reducing the dimensions and enabling a single-sensor system [19]. Like previous configurations, the number of beams is still limited due to the cumulative transmission loss. As the system described by Basiji and Ortyn [19] requires some lenses to disperse and direct the beams, a stack of tilted filters can eliminate the dispersive lenses and further reduce the size, as illustrated by Ortyn *et al.* [20]. However, the filters operate in double-pass mode and, therefore, prevent the increasing of the number of bands. A different strategy takes advantage of multiplexed volume holographic gratings written in a thermally-stable photosensitive glass [21]. A 12-channel beam splitter is prototyped by multiplexing three volume holograms in a 2 × 2 array, and another prototype is planned to cover both the visible and short wave infrared (SWIR) regions.

Although beam splitting (see Figure 4) enables a snapshot MSI solution, there are a few limiting factors, such as the number of bands and the incident angle, apart from the space requirement and the high cost. In practice, multispectral beam splitting has been applied to cell and particle analysis used in biological and medical applications, such as cytometry [19,20].

#### 2.1.4. Interferometer-Based Techniques

From the beginning of spectroscopy, the interferometer has been one of the key components extensively used in spectral imaging devices. Among the variety of interferometers proposed, some of them have been adapted to MSI.

Kudenov *et al.* [22,23] designed an extension of the dispersion compensated polarization Sagnac interferometer (PSI) by including two diffraction gratings in a standard PSI. As a result, unique spectral passbands are amplitude modulated onto coincident carrier frequencies. Later, modulated multispectral images can be extracted in the Fourier domain. The main disadvantage of this approach lies in the optical design that utilizes only one dimension in the Fourier space and ties the spectral bands to the grating's orders.

Gorman *et al.* [24] describe a generalized Lyot filter that employs multiple cascaded birefringent interferometers to simultaneously spectrally filter and demultiplex multiple spectral images onto a single detector array An example of an eight-band multispectral image sequence is obtained without further processing. More spectral channels, however, require larger polarizers and may be affected by chromatic aberrations, which may limit this approach to 16 spectral bands [6].

#### 2.1.5. Filtered Lenslet Array

**Figure 5.**
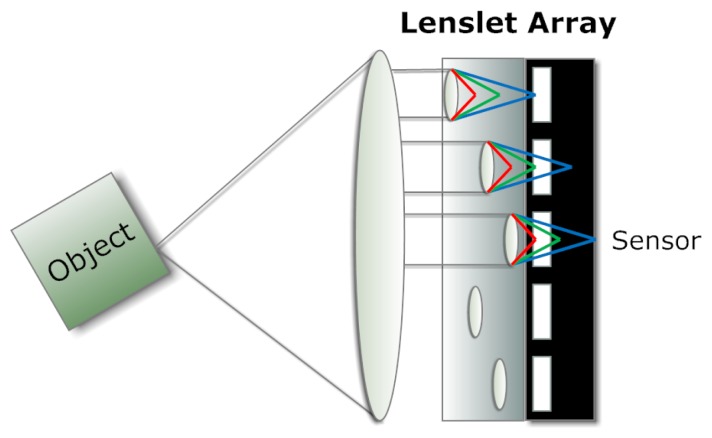
Example of a lenslet array, where the wavelengths are selected according to the distance between each lens and the corresponding photodetector.

As in a plenoptic camera, a lenslet array permits simultaneous observations of a point on the object by multiple photodetectors. Similarly, a filter array inserted in front of the lenslet array or image sensor enables observations of the spectra of the object when light passing through each lenslet is projected merely to the corresponding area on the sensor and the observation distance is set properly [25]. Pixels in the captured image are geometrically rearranged onto a multi-channel virtual image plane in order to reconstruct the multispectral image. The resolution of the resultant images is slightly reduced in comparison with the captured sensor image. Although simple in concept (see Figure 5), this approach requires fairly uniform irradiance in terms of angular distribution so that each filter and the corresponding lenslet capture similar power of light. That implies a limitation in the silicon sensor size, thus limiting the resolution.

Since this technique combines a plenoptic camera and a filter array, it may introduce multispectral information to all applications where an estimate of depth information is expected, such as the capture of 3D space [26], measurement of the oral cavity [27] and thermography [28].

#### 2.1.6. Tunable Sensor

The development of tunable sensors is required, as it will eliminate the necessity of using external dispersive or wavelength selective elements, thus reducing the size and simplifying the complexity of an MSI system.

**Figure 6.**
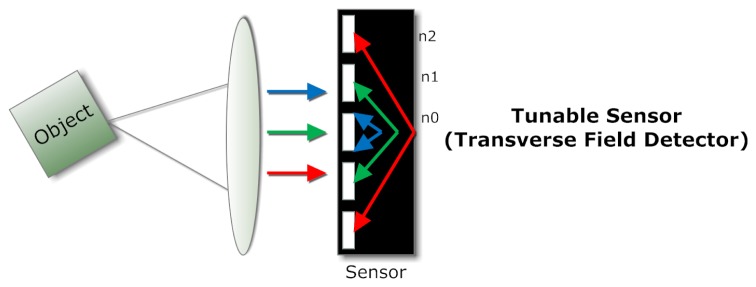
The multispectral reconstruction capability is based on suitable biasing. Carriers are collected by the contact pairs from different depths, and each contact pair has its own spectral response.

The transverse field detector (TFD) [29] is such a photosensitive device that exhibits different spectral responsivities at different depths by applying suitably biased voltage. Figure 6 shows this principle. An analysis of six-band TFD for MSI is carried out [30], and one of the advantages of a TFD is the ability to tune the spectral responses on a pixel to pixel basis. Krapf *et al.* [31] demonstrated quantum-well infrared photodetectors (QWIP) for the purpose of multispectral infrared imaging application.

#### 2.1.7. Multispectral Filter Array Approach (MSFA)

The wide application of CFA [7] in color imaging has aroused academic [32] and industrial [33] interest in extending CFA to MSFA by integrating more types of filter elements into the mosaic. Similar to a CFA-based color imaging system, an MSI employing MSFA might also need to be the result of an optimization between spatial and spectral features. Moreover, it also needs the demosaicing process and might be affected by chromatic aberrations. The approaches falling into this category are detailed in Section 2.

#### 2.1.8. Hybrid Solutions

The aforementioned approaches to MSI have their pros and cons, and a hybrid system may well overcome some drawbacks while still maintaining the advantages.

Murakami *et al.* [34] suggest a hybrid-solution MSI device by merging a four-channel beam splitter with filtered and mosaicked sensors. Conceptually, this is equivalent to three R/G/B images of high resolution coupled with an MSFA mosaic image of low resolution. The image is then reconstructed on the basis of small regions by linearly combining the regions in the R/G/B band images with the weighting coefficients determined from MSFA data.

Skauli *et al.* recently presented a new spectral sensor concept that integrates a few filter stripes in the focal plane [35]. When scanning the field of view, the filters of six bands provide spectral information, while the remaining photosensitive area still captures normal 2D monochromatic images.

Tamburrino *et al.* [36] integrated CFA and the stacked photodiode structure of a CMOS image sensor. The red and blue filter elements in the original Bayer pattern are replaced with green-absorptive magenta filters under which lies the two stacked and pinned diodes that mostly absorb blue and red light. Similarly, Martínez *et al.* [37] combined CFA with TFD to narrow down the bandwidth of TFDs and improve the accuracy of spectral and color reproduction. Yet, another idea is presented by Sparks and DeWeert [38], where a vertically-staked multi-layer sensor, known as a Foveon sensor [39], is covered by a two-band filter array consisting of two types of triple bandpass filters, which forms a six-band MSI sensor.

### 2.2. Multispectral Filter Arrays

Unlike CFA mosaic design, which mostly incorporates three types of primary color filters (The differentiation between CFAs and MSFAs does not lie in a clear number of bands: mostly CFAs include three to four bands, while MSFA might include four to a high number of bands. However, up to now, we might consider that a CFA aims at the retrieval of relative color information, whether an MSFA aims at object property estimation and/or objective color measurement), the number of bands in an MSFA may vary a lot, and the choice of filters can be rather specific to the application. In recent years, there have been quite a few proposals for MSFA mosaic patterns, as well as the methodology of design. However, due to its versatility, the manufacturing difficulties and cost, there have not been too many practical industrial implementations of this solution.

#### 2.2.1. MSFA Design

To the best of our knowledge, Ramanath *et al.* first presented a modified CFA for multispectral image acquisition [32]. Seven types of filter elements in this MSFA are arranged hexagonally, such that each pixel of a certain spectral band is surrounded by six pixels of distinct bands. As a result, the demosaicing for each pixel may be performed with the nearest neighborhood interpolation. This idea is further detailed in [40] along with the techniques in designing spectral sensitivities for the sake of recognition and reconstruction.

Miao *et al.* put forward a generic method of MSFA design where the spectral bands' probability of appearance in the array can be represented in a binary tree [41,42]. It starts from a checkerboard pattern and further splits the pattern into children by the power of 1/2 following the binary-tree. An accompanying generic demosaicing algorithm is also developed [43,44]. When used in sequence, these two proposals complement each other and are the very first systematic attempts at MSFA-based MSI [45]. Also presented in [42] is a framework of the evaluation of MSFA design, and another similar quality metric is explained in [46].

In contrast to a complex pattern designed with, e.g., the binary-tree approach, Brauers and Aach [47] propose a six-band MSFA arranged in 3 × 2 moxels in a straightforward manner, which is aimed at faster linear interpolation. Another simple MSFA is from Aggarwal and Majumdar, who arranged four filters in diagonal stripes [48].

The spectral sensitivity of solid-state photodetectors ranges from ultraviolet through the visible region, all the way to LWIR (long wavelength infrared). Thus, Hershey and Zhang [49] designed a camera integrating both visible light and non-visible light photodetectors in a single MSFA. In fact, the mosaic is mostly the same as the Bayer pattern, except one green in the 2×2 moxels is replaced with a non-visible filter element.

MSFA is essentially a spatio-spectral sampling mechanism. When consisting of sufficient types of narrowband filter elements, an MSFA mounted sensor can be used as a spectrometer. Wang *et al.* designed an 8 × 16 MSFA that comprises 128 distinct narrow bandpass filters for capturing NIR (near-infrared) spectra [50].

In remote sensing applications, the band of LWIR plays an important role in material identification. In contrast to the conventional line-scan MSI sensor, Mercier *et al.* examined the usefulness of an MSFA snapshot LWIR sensor [51]. Both the optimal number and width of the spectral channels are analyzed with simulated typical background signals.

For the purpose of jointly capturing RGB and NIR images, Lu *et al.* formulated the design of MSFA as an optimization problem in the spatial domain [52] and provided an iterative procedure to search for locally optimal solutions, considering that the spectral sensitivity of modern solid-state image sensors extends from the visible range to the NIR region. The resulting mosaic pattern consists of 16 bandpass filters arranged in 4 × 4 moxels, 15 of which are visible, and one is NIR. An improved algorithm is later developed that takes into account the correlation between visible and NIR bands, where the optimization problem is addressed by mean of regularization [53]. Results obtained via the analysis of multispectral joint visible and NIR video for background removal [54] show that it might be of high benefit in robotics.

Following the concept of a generalized assorted pixel (GAP) camera where post-capture adjustment can find the best compromise among spatial resolution, spectral resolution and dynamic range, Yasuma *et al.* designed a seven-band MSFA composed of three primary-color filters and four secondary color filters [55].

Monno *et al.* proposed a five-band MSFA [56]. In the pattern, the green-like channel is distributed in the form of a quincunx, as in the Bayer CFA. Other channels are arranged following the binary-tree approach [41], so that the adaptive kernel can be estimated directly from the raw data for the purpose of subsequent demosaicing.

The spatial arrangement of the filter elements plays an important role in MSFA compared to in CFA, as reported by Shrestha and Hardeberg [57]. It has been found that the influence of the mosaic layout tends to be more prominent as the number of bands increases, *i.e.*, as the distance between spectrally similar pixels increases. Further, the authors show a particular MSFA pattern aimed at both spectral reconstruction and illuminant estimation, which is an instance generated by the binary-tree approach [41].

To integrate one more band, *i.e.*, IR or UV, into a common CFA and to maintain its compatibility with the CFA maximally, Kiku *et al.* proposed a modified Bayer pattern where the additional band is sparsely sampled and the filter elements are arrayed on a slightly slanted square grid [58].

To verify the usefulness of compressive sensing (CS) in MSFA demosaicing, Aggarwal and Majumdar present two five-band MSFAs [59]. One of them is a random pattern where each channel has equal probability of appearance. Another one is a uniform filter array similar to the one proposed in [48]. In theory, such uniform sampling patterns are not conducive to CS recovery, so it is experimented with for comparative purposes only. Both of the two MSFAs are easily extendible to any number of channels.

From this state-of-the-art, it appears that there are many possibilities to design an MSFA sensor. Intuitively, we can already understand that given the variety of design, data processing will be very dependent and the cost will be high while designing specific arrangements. All hardware implementations of multispectral filters offer solutions with some issues, such as the lack of sensitivity, crossleak problems between channels, inaccuracy measurements, or do not consider the overall system characterization (sensor + filters). As indicated by the state-of-the-art, it is not difficult to find out that the design of MSFA is rather application-specific in terms of the number of bands, spectral transmittances, as well as the geometrical arrangement. It is therefore a must to keep the subsequent processing in mind and to consider all of these as a whole. Furthermore, the cost of MSFA would be acceptable only in the case of sufficient production volume.

**Figure 7.**
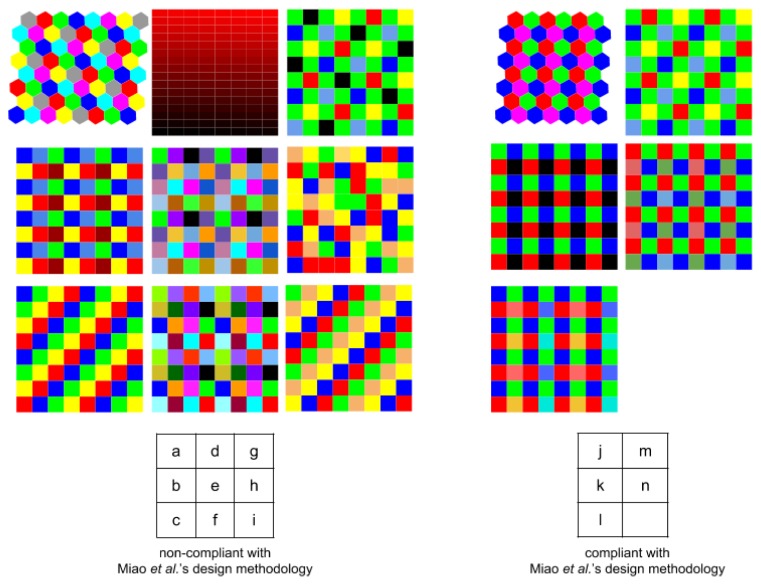
**(a)** Ramanath *et al.* [32]; (**b**) Brauers and Aach [47]; (**c**) Aggarwal and Majumbar [48]; (**d**) Wang *et al.* [50]; (**e**) Lu *et al.* [52]; (**f**) Sadeghipoor *et al.* [53]; (**g**) Kiku *et al.* [58]; (**h**) Aggarwal and Majumbar [59]; (**i**) Aggarwal and Majumbar [59]; (**j**) Ramanath *et al.* [32]; (**k**) Hershey and Zhang [49]; (**l**) Yasuma *et al.* [55]; (**m**) Monno *et al.* [56], Shrestha and Hardeberg [57].

We present in Figure 7 a list of the spatial arrangements from the state-of-the-art, classified between *ad hoc* design or compliant with Miao *et al.* [43,44].

### 2.3. Practical Realization of MSFA

MSFA has not been as widely accepted by the industrial community as CFA yet. Among other difficulties, the production and fabrication of MSFA present a major technical challenge. Sustained effort, therefore, went into realizing the MSFA.

In [33], a production process is presented where a dichroic filter array can be produced on a wafer and later bonded to an image sensor for the purpose of spectroscopic imaging. Dichroic filters, also known as interference filters, enable custom filters with spectrally sharp transitions, thus a better selectivity of color. A compact sensor with a lithographically patterned dichroic filter array is presented [60–63] where, at most, 10 wavelength bands can be incorporated.

A four-band MSFA sensor dedicated to medical applications is described by Sprigle *et al.* [64]. The optical filter consists of four narrow-band cells at 540, 577, 650 and 970 nm and is fabricated with traditional multi-film vacuum deposition and modern micro-lithography technologies [65]. Further, a means of evaluating the spectral interference between adjacent channels is also developed [66]. Further information and applications are detailed later in a series of articles concerning the detection of erythema and bruising, which are important for the prevention and diagnosis of pressure ulcers [67–70]. A paper by Qi [71] shows an implementation using a conventional Aptina sensor, where due to the hard manufacturing process, each cell of the filter cover 16 pixels of the full raw image. They propose software processing in order to avoid two types of degradations: the misalignment between the filter and the sensor and the reconstruction of missing spectral components (demosaicing). The implementation results show some success and promise a real-time production of multispectral images that allows instant detection.

To fabricate 128-band MSFA [50], Wang *et al.* developed a technique named combinatorial deposition [72] that combines the techniques of deposition and etching in order to produce spacer arrays with the different thicknesses required by the corresponding Fabry–Pérot-type filter element. Such a device makes possible *in situ* spectral measurement of NIR spectra ranging from 722 nm to 880 nm. Walls *et al.* designed, fabricated and characterized a 23-band MSFA of narrowband Fabry–Pérot filters with FWHM (full-width half-maximums) of 22–46 nm, covering the visible range (400–750 nm) [73]. The fabrication is suitable for direct integration onto CMOS image sensors in industrial foundries, and the cost and complexity is reduced in comparison with other solutions that vary the physical cavity length only. Another Fabry–Pérot interferometer-based snapshot multispectral sensor is developed by Gupta *et al.* [74]. The sensor employs a 16-band MSFA arranged in 4 × 4 moxels that operate in the SWIR (short wavelength infrared) range from 1487 to 1769 nm with a spectral bandpass of about 10 nm. The MSFA is installed in a commercial handheld InGaAs camera coupled with a customized micro-lens array with telecentric imaging performance in each of the 16 channels.

Geelen *et al.* introduced an MSFA sensor integrating tiled filters and optical duplication [75]. It is demonstrated that a prototype camera can acquire 32-band multispectral images of 256 × 256 pixels in the spectral range of 600–1000 nm at a speed of about 30 images per second in daylight conditions and up to 340 images per second in typical machine vision applications of higher illumination levels. Later, Geelen *et al.* proposed another MSFA imager by depositing interference filters per pixel directly on a CMOS image sensor [76]. The monolithic deposition leads to a high degree of design flexibility, so that an application-specific compromise between spatial and spectral resolution can be achieved.

**Figure 8.**
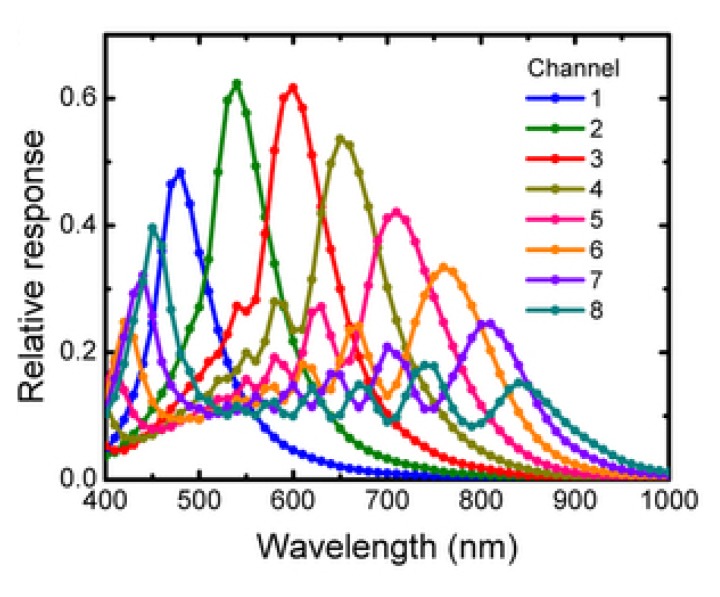
Relative response of multispectral imaging using vertical silicon nanowire photodetectors [77,78]. Reproduced here with their permission.

A significant study conducted at Harvard University [77,78] offered multispectral mosaicked filters based on nanowires. A wavelength-selective coupling to the guided nanowire mode is used in order to capture eight multispectral images from visible to NIR wavelengths. A polydimethylsiloxane (PDMS) film is mounted directly on a CCD monochrome sensor. The actual relative response of their system is presented in Figure 8. They show particular image experiments dedicated to Normalized Difference Vegetation Index imaging.

### 2.4. Adequate Processing: Demosaicing

One aspect not very well developed in the existing implementations, but still a very strong limitation of MSFA technologies, is the need of specific processing that takes into account the design and technology used. Indeed, if there is not a demosaicing process associated with the design, the loss of resolution might be critical. Moreover, it seems that simply extending CFA demosaicing methods will not give the best results depending on filters and distributions [79]. Methods for demosaicing MSFA images have been explored in recent works, which are presented below.

Along with the MSFA generation method, an accompanying generic demosaicing algorithm was also developed by Miao *et al.* [43,44], which interpolates each band independently by tracing the same binary tree back. The interpolation is edge directed and performed level by level following the binary tree.

To interpolate the mosaic image associated with the MSFA presented in [47], Brauers and Aach advanced a demosaicing algorithm where channel difference is first smoothed before being linearly interpolated.

Following the binary-tree approach [43,44], Baone and Qi posed demosaicing as an image restoration problem and address it with the non-linear maximum *a posteriori* (MAP) probability technique using the gradient descent optimization process, for images mosaicked by a seven-band MSFA.

Lu *et al.* came up with the linear minimum mean square error (LMMSE) approach to the joint demosaicing of RGB and NIR images [52], formulating demosaicing as an image restoration problem. In this case, the objective of MSFA design is meant to provide the minimum reconstruction error in terms of LMMSE.

To reconstruct multispectral images from GAP mosaicked sensor output, Yasuma *et al.* came up with a multimodal image reconstruction framework where primary and secondary color images are reconstructed separately [55]. The former is demosaiced by means of low-pass filtering in the Fourier domain, since the sampling rate is relatively high. Therefore, the demosaicing of the secondary spectral bands, which are less sampled, exploits the inter-channel correlation between the most similar primary and secondary filter pair, in the principle of constant channel difference and residual interpolation.

Having designed the five-band MSFA keeping interpolation in mind, Monno *et al.* introduced adaptive kernel upsampling to MSFA demosaicing [56]. The proposed adaptive Gaussian upsampling (A-GU) and joint bilateral upsampling (A-JBU) are extended from the corresponding non-adaptive methods, respectively. The adaptive kernel is estimated directly from the mosaic image, which is first used by the A-GU to generate a guide image from the green-like band for the A-JBU. After that, A-JBU, with the same adaptive kernel, is applied to each of four other spectral bands. Later, Monno *et al.* replaced the A-JBU with the guided filter [80], known as an edge-preserving filter that also requires and depends much on the guide image. Recently, Kiku *et al.* adapted this means for the demosaicing of the hybrid MSFA pattern [58]. The sparsely sampled additional band is separately interpolated with a super-resolution technique with the sparse mixing estimators, whereas the R/G/B channels are interpolated following basically the framework described in [80] with a few improvements, including a newly proposed gradient-based interpolation of the green channel, as well as an iterative procedure that samples the reconstructed image as the input to the following iteration.

In search of general MSFA demosaicing techniques, Wang *et al.* first extended vector median filtering demosaicing [81] to the multispectral domain [82]. The nature of the vector median filter ensures that the results of filtering are derived from input vectors, namely the filtering does not introduce new values to the vectors, but only interpolates a missing value at one band with another value in the vicinity at the same or another band. In light of the number of bands in an MSFA, this still produces visible artifacts; therefore, the authors also complemented this approach by a subsequent refinement step. Later, Wang *et al.* investigated the use of discrete wavelet transform in MSFA demosaicing [79], following Kim *et al.*'s work [83]. It operates in the wavelet domain, and the low-frequency and high-frequency components are interpolated differently. To benefit from the inter-channel correlation, the high-frequency components of an unknown band are replaced with the known values of another band, while the low-frequency bands are interpolated individually and linearly. As a result, the performance of such a technique depends a lot on the inter-channel correlation. Recently, their effort went into combining linear MMSE and residual interpolation [84]. The linear MMSE between the original and the reconstructed images is achieved by the Wiener estimation. Next, the difference between the interpolated images and the original image is derived, *i.e.*, the residual is interpolated, so as to complete the demosaicing.

Aggarwal *et al.* put forward a series of MSFA demosaicing methods. The first represents a pixel in question, also a central pixel in a given neighborhood, as a linear combination of neighboring intensity values from the same and other bands [48,85]. In other words, the linear filtering is performed on the raw mosaic image with a given kernels whose parameters may be determined by means of training [86]. Recently, the authors attacked MSFA demosaicing with compressive sensing [59], where both group-sparse reconstruction and the Kronecker compressed sensing are explored. The results demonstrate that the latter method outperforms the former, and the random pattern always yields better results in both approaches, except that the uniform pattern does a better job in the Kronecker method for three-band demosaicing.

Dealing with a SWIR sensor coupled with a nine-band MSFA filled by 3 × 3 moxels, Kanaev *et al.* are confronted with two difficulties in demosaicing: first, the inter-channel correlation here is not usable; second, the distribution of each band is equal to another, so there is no comparatively oversampled channel. To overcome these two drawbacks, the authors introduce two approaches to demosaicing. One makes use of the multi-band edge information, while the other applies multi-frame super-resolution to the enhancement of multi-spectral spatially multiplexed images [87].

Due to the huge number of possibilities in the design of MSFAs, there exists and there will exist a huge number of possibilities for processing. It appears that if MSFA might be application dependent, a very good compromise might be created in combining the spatial distribution, the demosaicing and the spectral definition of the moxel. However, to handle this problem, there is still the need of a unified mathematical framework.

## 3. Practical Implementation

Ultimately, we are interested in evaluating demosaicing algorithms in practice. However, it is very difficult today to obtain an MSI system based on MSFAs from the standard market. We are aware of a few prototypes that are jealously kept away by their owner, either due to industrial secrets (Olympus, Canon, *etc.*) or academic research advancement reasons. We thus decided to implement our own sensor from commercial elements. We wish this section to be useful to any researcher or industry who may want to design its own sensor. This section describes the elements we combine in order to obtain such a sensor.

In many cases, the NIR channel is also of benefit in image processing, either for color image enhancement (e.g., skin texture improvement) or for robotic vision (e.g., shadow removal). Silicon sensors typically respond to incident radiation in the visible and NIR range of the spectrum. Thus, to keep the generality of the sensor, we design MSFAs to capture wavelengths between 400 nm and 1100 nm in the visible range and the NIR range. Our solution combines a standard sensor built on CMOS technology, with customized filters. This section presents the spectral and spatial characteristics of these two components.

### 3.1. The CMOS Sensor

**Figure 9.**
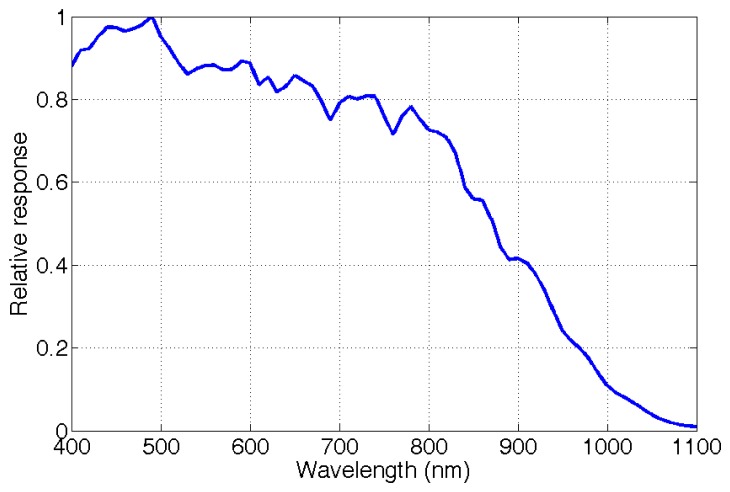
Relative response of the e2v EV76C661ABT sensor [88]. The measurements were done using the OL Series 750 Spectroradiometric Measurement System [89] with a tungsten lamp.

The sensor is a CMOS Sapphire EV76C661 from E2V [88]. It offers a 10-bit digital readout speed at 60 frames per second (fps) with full resolution. This sensor provides relatively good sensitivity in the NIR spectrum (quantum efficiency > 50% at 860 nm), while keeping good performance in the visible spectrum (> 80%). Due to the generally relatively low transmission factors of the filters, it is important to have good pixel sensor quantum efficiency. This can tolerate more noise and is favorable for low-light sensing. The sensor also embeds some basic image processing functions, such as image histograms, defective pixel correction, evaluation of the number of low and high saturated pixels, *etc*. Each frame can be delivered with the results of these functions encoded in the image data stream header. The resolution of the sensor is 1280 × 1024, and each pixel has an area of 5.3 squared micrometers. We measured its sensitivity with a monochromator (OL Series 750 Spectroradiometric measurement system [89]) by sweeping the wavelength of the light from the monochromator from 400 nm to 1100 nm in steps of 10 nm. A tungsten light source is used as a tunable light source. We can establish a trial characterization of the sensor and evaluate the SNR compared with the theoretical curves that we have simulated. The power supply of the light source and the wavelength of the monochromator are controlled by a computer. The sensor is used without any lens mounted in front of the camera. The formula used to recover the quantum efficiency (*Q_E_*) is shown in Equation (2):

(2) $$Q_{E}\left(\lambda \right)=\frac{hc\times D_{i}\left(\lambda \right)}{I\left(\lambda \right)\times \lambda \times \Delta t\times S_{e2v}}$$

where *h* is the Planck constant, *c* the speed of light in vacuum, Δ*t* the exposure time used for characterization, *D_i_* the digital intensity, *I* the irradiance at a specific wavelength and *S_e_*_2_*_v_* the area of an effective sensor pixel. The pixel values from the image captured by the e2v sensor at each wavelength are recorded. We find the relative sensor response, which is shown in Figure 9.

### 3.2. Filter Design

Filter design is not a trivial task. There are several causes for this, including the different aspects of the manufacturing process, sensing constraints and applications. Indeed, the optimal transmittances may be different depending on whether color acquisition or spectral reconstruction is considered. For a specific application, different bands might need to be acquired accurately. Moreover, demosaicing performance depends on the transmittance, as well.

Usually, the design of an MSFA begins with filter design. The literature addresses these problems as the definition of an optimal set of filters. In the context of color and multispectral imaging, the question of filter selection and optimization has been well studied [90–94]. Merely a few of them, however, take MSFA into account and concern filter design optimized for demosaicing [95], for spectral reconstruction [96], for energy balance [97] and for a combination of high dynamic range, color and spectral reproduction in a generalized assorted pixel camera [55]. However, these optimized filters are often based on simple theoretical transmittance function, *i.e.*, Gaussian or Gaussian-based curves.

On the other hand, the results of the optimization processes cannot yet be taken as they are. There is a major difference between the definition of the optimal set and what is available as commercial filters, due to manufacturing processes. There is no doubt that in the next decade, this problem will be bypassed with the advancement of technology, but it is still a limiting problem today. Nevertheless, one may use a brute approach to select the best filter set within a database [98]. This is valid in the case of large-sized filters that cover the sensor, such as a filter wheel.

When it comes to the problem of having a mosaic of filters, the technological constraint is even worse. There are only a few manufacturers that can realize such a mosaic. The large companies that manufacture CFAs master the technology, but they usually do not have more than a few numbers of filters on hand and mostly do not want to produce a small number of sensors. Even 10,000 items are barely of interest to them. Medium manufacturers, such as Ocean Optics, might be interested in realizing such sensors for 10,000 items, but they would be far too expensive for 10 prototypes. The third choice is small start-up companies in niche markets. They mostly master only one type of process, and then, the choice of filters is very reduced.

On top of these constraints, one may have a noticeable difference between the simulation and the experimental realization. These constraints need to be addressed; we wish to converge to something better in a few years.

While keeping these problems in mind, we design our filters in a very pragmatic way in partnership with a start-up company and using as much as we could of their existing expertise.

Our customized matrix of filters is built by SILIOS technologies [99]. SILIOS Technologies developed the COLOR SHADES^®^ technology, allowing the manufacture of transmittance multispectral filters. COLOR SHADES^®^ technology is based on the combination of thin film deposition and micro-/nano-etching processes onto a fused silica substrate. Standard micro-photolithography steps are used to define the cell geometry of the multispectral filter. COLOR SHADES^®^ provides band pass filters originally in the visible range from 400 nm to 700 nm. Through our collaboration, SILIOS developed filters in the NIR range, combining their technology with a classical thin layer interference technology to realize our filters.

**Figure 10.**
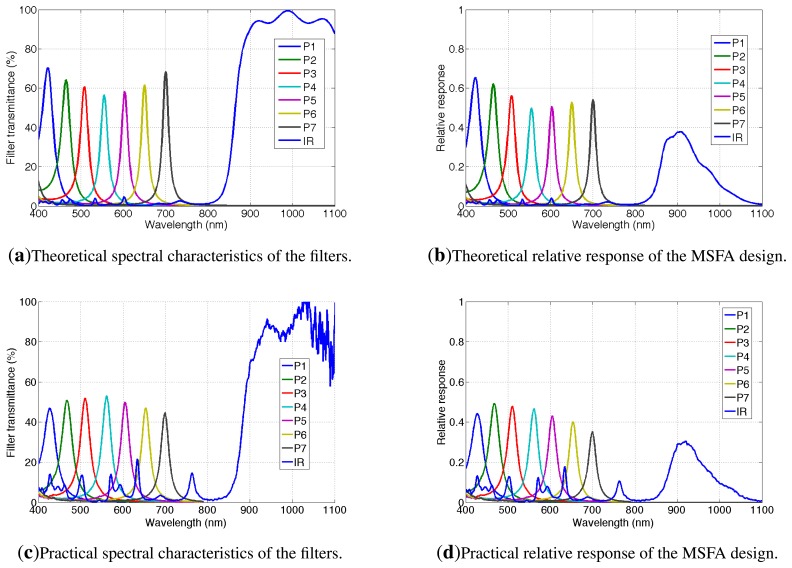
(**a**) Spectral characteristics of the filters. Channels {*P*1 – *P*7; *IR*} are labeled following the scheme of Figure 11; (**b**) Simulation of the relative actual response of the multispectral imaging system (sensor associated with filters). Measured data (**c**) and (**d**) highlight some spectral differences compared to simulation.

The transmittance of the eight sets of wavelengths responding to the light going through our filters is shown in Figure 10a. These curves are based on simulation and are provided by the manufacturer. The filters are for eight bands, {*P*1, *P*2, *P*3, *P*4, *P*5, *P*6, *P*7, *IR*}. The spectral characteristics of the filters (simulated and real) are shown in Table 2. We chose to present the central wavelength, the maximum transmission and to show the spectral difference between two wavelengths at half maximum (FWHM). The effective band is calculated for a given filter, taking bandwidth with transmission over 5% of the maximum transmission *T_max_*. The NIR filter has a specific shape, since it is based on classical thin layer interference filters. The global shape of this filter is an addition of Gaussian-like curves. The rising-edge of the high-pass NIR filter is located between 850 and 900 nm.

Figure 10c shows the transmittance of the actual filters measured with a monochromator. Compared to the theoretical responses expected in Figure 10a, we can see that the maximum transmittances decrease in the extreme parts of the visible spectrum. The peak sensitivities did not move critically. Two major differences appear on the IR spectral response:

1. The IR increasing front is centered on 885 nm instead of 865 nm ;
2. The IR rejection is worse than expected with one peak at 20% and four peaks at 10% in the visible range. The transmittance in the visible range is due to manufacturing problems. Indeed, the multilayer process is very complicated on areas of a few microns, and parasites and inefficient areas can occur.

The very bad cut off in the visible range for our IR filter will impact the sensitivity of the final sensor in two ways: First, the IR channel will contain visible information if there is no post-processing involved. However, since we have information within the visible range, up to 780 nm, it is possible to include a software dynamic correction to the IR channel, which will then contain information captured only along the last part of the filter. Second, the energy balance of the sensor can be critically affected.

Figure 10b,d shows the MSFA sensor sensitivities (simulation and actually measured), which combine the CMOS sensor and our filters.

#### 3.2.1. Energy Balance

To test the energy balance of our sensor [97] and to evaluate its ability to acquire multispectral information in one single shoot, we compute the convolution between illumination, filters and sensor spectral characteristics, such as described in Equation (3):

(3) $$\rho_{p}=\int_{400}^{1100}\text{Illuminant}.R_{e2v}.T_{\lambda ,p}d\lambda$$

where *p* ∈ {*P*1 – *P*7, *IR*}, *R_e_*_2_*_v_* is the relative response of the single sensor and *T_λ,p_*, is the transmittance of each filter *p*. The convolution results are normalized with the maximum transmittance in the visible range for each illuminant. The result is shown in Table 3. We note that the energetic distribution is reasonably balanced in the visible range in natural exposures, since the variance between the spectral bands is acceptable for illuminants *E* and *D*_65_. Results can be compared to the typical curves of the RGB Sinarback camera [100], where the convolution variance is empirically considered to be good enough for the sensor energy balance for an RGB device. It is likely that a single exposure is sufficient to capture bands P1–P7. Exposure setting tests will probably confirm this analysis in future works.

Due to the high sensitivity of the NIR component, some scenes captured could be represented by over-exposed in the NIR or under-exposed pixels in the visible multispectral image. Taking into account the intended application, one can imagine the use of a less sensitive sensor in the infrared or the use of a simple low-pass filter added to the camera lens. This would reduce the sensitivity range of the IR pixel, but would improve the energy distribution of the light on all of the sensor bands. Illumination influences greatly this analysis, for instance it is more likely that with an illuminant of Type A, this sensor would be unbalanced and that a specific low pass filter would be of benefit to the acquisition.

#### 3.2.2. Spectral Interference

Looking at Figure 10a,c, significant overlapping areas among spectral bands can be noticed. There are mutual interferences that we may quantify. According to previous works [66], it might be reasonable to determine interference coefficients using this integral ratio:

(4) $$\Theta =\frac{\int_{\lambda a}^{\lambda c}T_{i}d\lambda +\int_{\lambda c}^{\lambda b}T_{j}d\lambda }{\int_{\lambda m}^{\lambda n}T_{j}d\lambda }$$

where *λa* and *λb* are the wavelength coverage between two filters, *λm* and *λn* are related to the effective band of filter *j* and *λc* is the wavelength at the intersection of the two filters. The indexes are presented in Table 4. We naturally note that the farther the filters are, the smaller the coefficient is. These values can be used to study the correlation of the results of spectral reconstruction and/or the demosaicing with the relative spectral interference. These indicators and other similar indicators are however difficult to interpret without quantitative data and statistics applied on a data set.

### 3.3. Spatial Arrangements

This section considers the size and spatial arrangements of the filter arrays.

**Figure 11.**
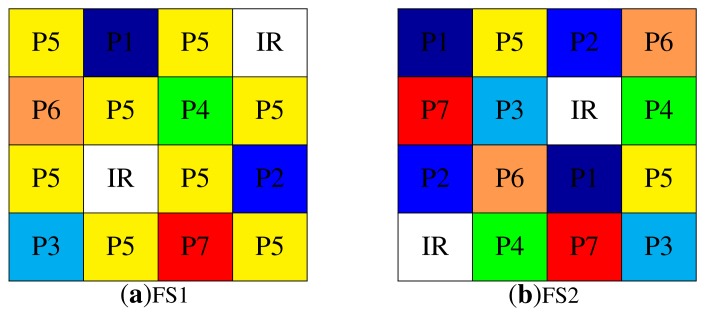
FS1 (**a**) and FS2 (**b**) are two different moxels. FS1 shows an *ad hoc* distribution with over-sampled channels (P5 and NIR), FS2 shows uniformly distributed samples as an instance of Miao *et al.*'s [44] binary tree algorithm. Refer to Figure 10c for the spectral characteristics of the filter channels.

In a mosaicked image, each pixel performs a direct measurement through one specific spectral band, and the unmeasured values are estimated by its closest neighbors. This requires that the filter array is distributed as evenly as possible for a better interpolation. The hypothesis of correlation between spatial positions of different spectral bands has to be taken into account. Arranging the MSFA pattern is a major challenge. Beside the method proposed by Miao *et al.* [44], the distributions are usually *ad hoc* or the result of an optimization process [101]. We defined two different periodic spatial distributions corresponding to two different approaches. One of them promotes the spatial information, while the other promotes the spectral information. The filter arrangements chosen are shown in Figure 11a,b.

FS1 offers over-sampled spatial information of two spectral bands. P5 is designed as the green channel in a Bayer CFA, and IR is double-sampled compared to the rest of the filters. Such an arrangement is supposed to provide a good spatial reconstruction and reasonably good information in the NIR domain. In doing that, we assume that the application might benefit from a good NIR and spatial knowledge, *i.e.*, joint visible and NIR dehazing, shadow removal or illuminant estimation.

FS2 is designed to have sub-sampled spatial information that benefits more important spectral information. This filter is designed following the method proposed by Miao *et al.* [44] with equal probability of occurrence of each channel. This pattern has the same sampling frequency in both the horizontal and vertical directions, but it has a frequency doubled for diagonal directions. These patterns represent the state-of-the-art standard methods.

An MSFA image *M_MSFA_*(*x*, *y*) can be represented by a mosaicked image, with only one channel per spatial location. Each sampled component *c_p_* is represented discretely mixing spectral (*λ*) and spatial (*x*, *y*) characteristics of the matrix. We could imagine the projection of sub-sampled values on a unit vector of equal dimension to the number of channels:

(5) $$M_{\text{MSFA}}\left(x,y\right)=\sum_{p}c_{p}\left(x,y\right)Z_{p}\left(x,y\right)$$

where *Z_p_*(*x*, *y*) are orthogonal functions of dimension P. They take the values one or zero if the p-channel is present or not at the location (x, y).

**Figure 12.**
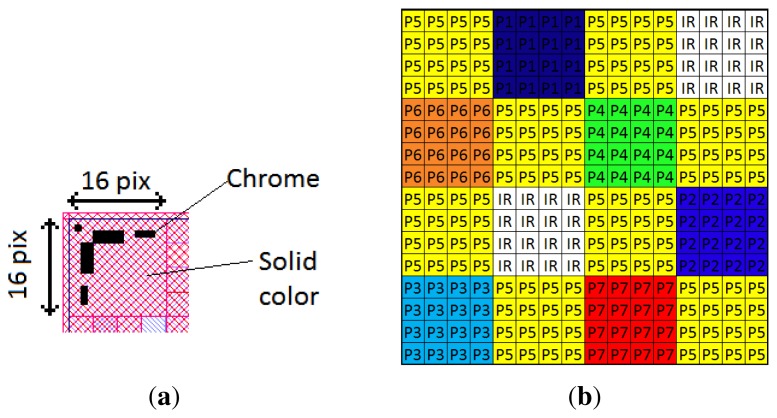
One alignment matrix (**a**), in the top left corner of the filter array; (**b**) Each filter covers 4 × 4 pixels of the sensor.

Each filter size of the mosaic should be ideally the same as the CMOS pixel size. However, the manufacturing difficulties and the physical limitations in achieving this level of spatial resolution would increase dramatically the cost of the product, if even feasible. In our practical case, the sensor pixel pitch is 5.3 μm, but each filter element measures 21.2 × 21.2 μm^2^, corresponding to 4 × 4 sensor pixels. The actual resolution of the mounted filter is then equal to 320 × 256 pixels (but the sensor is populated by 1280 × 1024 pixels). Figure 12b shows the real overlapping between sensor pixels and filters. The total filter matrix size is 6.78 × 5.43 mm^2^. Additionally, a margin is introduced in order to support mechanical switching during assembly; that is why the total carrier physical size is 6.9 × 5.6 mm^2^. Regarding the alignment and assembly of the filters with the sensor pixel matrix, alignment matrices are drawn in the corners of the filters. These areas occupy 16 × 16 CMOS pixels in each of the four corners of the physical matrix. These matrices are designed with solid color and chrome patterns for tracking. Figure 12a shows one of these matrices.

After fabrication, the MSFA is mounted on the sensor directly on the microlens array. It is necessary to remove the glass covering the sensor before the implementation of the filter array. The process of setting up the filter on the sensor is a very delicate operation. We will not describe the process here. A supplementary information paper discusses this aspect in details [78].

**Figure 13.**
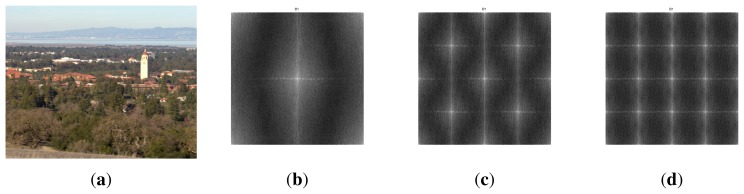
(**a**) The multispectral image by Skauli *et al.* [102] is used; (**b**,**c**,**d**) Log-magnitude representation of spatial arrangements for both FS1 and FS2. (**a**) Stanford image; (**b**) P5 over FS1; (**c**) IR over FS1 and P{1,2,3,4,5,6,7,IR} over FS2; (**d**) P{1,2,3,4,6,7} over FS1.

To test the geometric/algebraic structure of the sampling patterns of the multispectral filter arrays FS1 and FS2, we analyze spatial subsampling through a log-magnitude representation of the Fourier transform using the Skauli Stanford Tower image [102] (see Figure 13). The radiance data cover wavelengths from 415 nm to 950 nm in steps of approximately 3.65 nm, spanning the visible and NIR spectral ranges. The image is processed as it simply simulates an acquisition by our sensor, with negligible optical effects. We compute the amplitude frequency spectrum of a single-channel per pixel sub-sampled image. This representation allows us to clearly visualize the spatial frequency representation. Figure 13b illustrates the fact that we have one pixel in two (vertically and horizontally) on the P5 channel of FS1 pattern (Nyquist frequency). Figure 13c shows that we have one pixel in two in the diagonal for most of the channels. In Figure 13d, we can observe the sparser sampling of most spectral channels of the FS1 pattern, one sample every four pixels in all eight directions. The Fourier representation helps to anticipate some problems of aliasing that will arise during the reconstruction process of images from a given MSFA pattern.

#### 3.3.1. Pixel Alignment

Many optical systems exhibit cross-talk phenomena. Some photons can be intercepted by an adjacent pixel than the one for which they were intended. This can contaminate the adjacent pixels and lead to some artifacts. The phenomenon becomes more and more common since the industry tends to shrink the device footprint, because the pixel sensors are more densely packed together. This effect appears in the case where the filter is positioned below the microlens and is even more likely to appear in our case. Besides, there might be some inaccuracy in the positioning of the filter on top of the sensor, which will increase crosstalk. We can also anticipate artifacts in the manufacturing of the filters and fuzzy borders between pixels.

This phenomenon must be quantified in the near future. We can notice that in our case, crosstalk and chromatic aberrations could be less annoying since each filter covers an array of 16 pixels uniformly sensitive to a specific wavelength. This might cause less damage than a recurrent artifact, which interferes with the object recognition, for example.

### 3.4. System Integration

**Figure 14.**
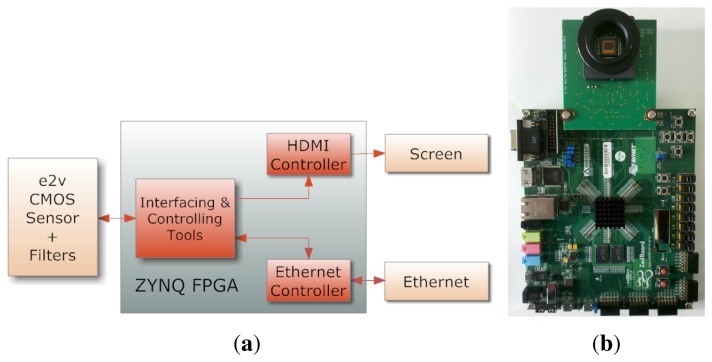
Overview of the hardware/software system integration, with a front view of the assembled camera without a lens (**b**); This camera architecture is used in order to test and characterize the sensors; (**a**) Our global system; (**b**) Zedboard + sensor daughter board.

An extension of a custom PCB board was designed. It is plugged into an FPGA board with Zynq (Zedboard), to support the EV76C661 image sensor. The customized electronic sensor board is designed at our lab (see Figure 14b). A part of the hardware description previously performed in our laboratory [103] was re-used (sensor configuration, video timing detection/generation and display). Since the sensor operates at 60 fps, we chose to implement the solution on an FPGA board. Opportunities for future hardware implementation of real-time image processing are then permitted. The whole system is presented in Figure 14. The HDMI controller permits a real-time visualization at a full SXGA resolution (1280×1024 pixels) at 60 fps. We can obtain a raw video at 10 bits/pixel without compression, using an Ethernet communication running at 120 Mbits/s. The frame rate for receiving video through Ethernet is about 17 fps.

**Figure 15.**
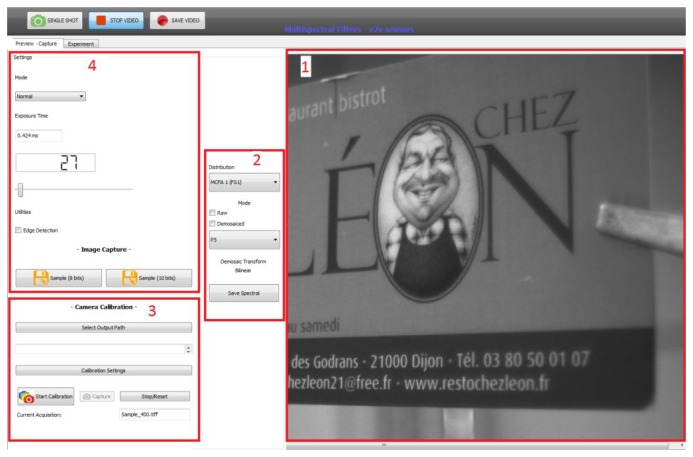
The control software. Application features: 1, video preview; 2, channel and filter selection; 3, characterization tool; 4, setting tools. A preview after bilinear demosaicing is a functionality of the application.

Software provides the control of most functions and the acquisition of images or video sequences (see Figure 15). This software is used to retrieve the video stream from the sensor through the UDPEthernet protocol. A live preview of the resulting image before and after demosaicing is also available for both arrangements FS1 and FS2. To ensure high speed and a real-time preview of the acquisition, only simple interpolation demosaicing has been implemented. We added the possibility of recording a TIFF image with multiple pages, each page corresponding to one channel. We can also save the arrangement of the filters in the metadata of the demosaiced/raw image. On Blocks 1 and 2, we can see the preview of the image acquired on a selected channel. This is very convenient for focusing on a specific filter depending on the pattern used (*i.e.*, the P5 channel of FS1). Block 4 permits us to configure the sensor (exposure time) and to perform video acquisition or a snapshot. Block 3 is a tool dedicated to the spectral calibration with a monochromator.

## 4. Prospective Work on Demosaicing

This section presents preliminary results on demosaicing and a comparison between the two setups, FS1 and FS2. We simulated our system in MATLAB, creating an environment able to represent all stages of the pipeline treatment. The simulation of the light source, the radiance calculation, the image mosaic reconstruction and the multispectral image reconstruction are the main steps of our simulation software.

Future work will focus on a full resolution channel interpolation, dedicated to these spatial arrangement and spectral characteristics. Indeed, we must consider this research to ensure better image quality. The reduced spatial image resolution is a typical problem of CFAs and MSFAs due to their intrinsic property that a certain spectral band is allocated specifically at each location of the array. The manufacturing process requires us to have 16 (4 × 4) adjacent photosensitive elements for one filter.

So far, our goal is not to build a perfect system with high resolution in the reconstructed images. We can either do a sub-sampling on each channel or keep the input resolution and perform a simple bilinear demosaicing. Our goal is to demonstrate the feasibility of the process. It is possible to obtain a multispectral video without using filter wheels or larger and more complex systems, only with a standard monochrome sensor and a filter placed above. In fact, some channels have better spatial resolution than others (FS1-P5, for example). That is why the next step of our work is to investigate demosaicing methods to improve multispectral image reconstruction, taking into account the relative benefits of our two filter arrangements.

**Figure 16.**
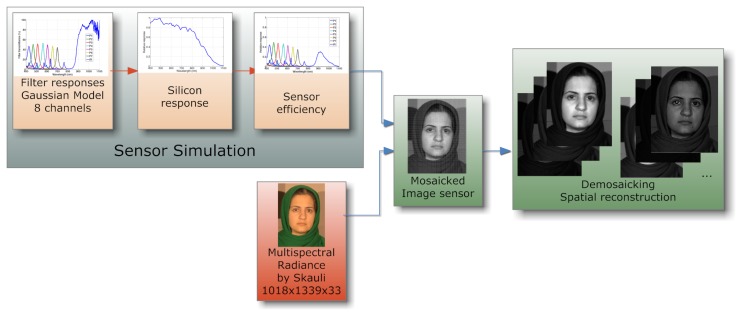
Simulation scheme of multispectral filters. The sensor system (photoreceptor and filters) is simulated using a particular illuminant and the silicon response of our sensor.

**Figure 17.**
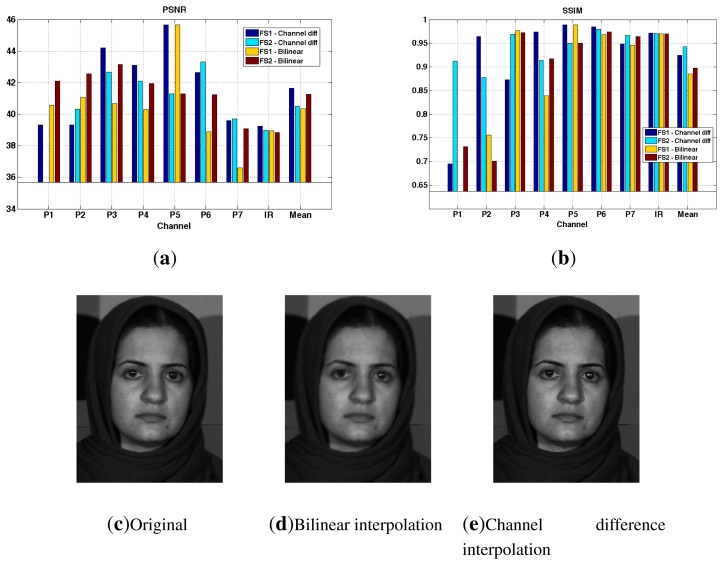
(**a**,**b**) Peak signal-to-noise ratio (PSNR) and structural similarity (SSIM) results of demosaicing simulation using the Skauli database [102]; (**c**) The ground truth image through the FS1 arrangement (P4 channel); (**d**) the image demosaiced by the bilinear interpolation and (**e**) the image demosaiced by the channel difference interpolation are shown.

Through the simulation of the system in MATLAB, we provide preliminary results on demosaicing from our two arrangements, FS1 and FS2. The demosaicing techniques used are bilinear interpolation and channel difference interpolation [104] (by the P5 channel). The simulation steps is shown in Figure 16, where we use the Skauli hyperspectral database [102]. We use standard indicators peak signal-to-noise ratio (PSNR) and structural similarity (SSIM) [105] to quantitatively measure the quality of reconstruction. These results are presented in Figure 17. We can see that PSNR for channel difference interpolation is better for FS1, due to the existing over-sampled channel (P5).

Figure 17c,d,e shows the results of the demosaicing following two methods for the P4 channel through FS1. Although PSNR and SSIM indicators do not show significant differences, it can be seen that the edges are significantly sharper for the image reconstructed by the channel interpolation method. This method is preferred for the FS1 arrangement.

Beside the obvious facts, interpretation of demosaicing on one image does not give a strong hint, and further work is required.

## 5. Conclusions

This paper presents a prototype of an MSI device using the innovative concept of MSFAs mixed with a commercial imaging sensor. It is suitable for hand-held and real-time imaging applications. We establish a consistent design of absorptive MSFAs, from elaboration to implementation. Two filters are implemented on a standard 1.3-megapixel sensor. The filters are constructed on a 2D substrate, so that different wavelengths of light can be captured simultaneously in snapshot imaging. Our simulation with several illuminants seems to indicate that it will not be necessary to use multiple exposure acquisition within the visible range, although doubts are present for the NIR part due to manufacture possibilities. Finally, the manufacturing process is relatively simple and reproducible. The resulting system is small compared to most existing multispectral vision systems.

However, the implemented solution will require some further investigations. We are particularly interested in the development of dedicated demosaicing algorithms. On the other hand, a huge amount of work is required to characterize the sensor and to investigate crosstalk effects among filter cells that will surely affect the results and performance.

## Figures and Tables

**Table 1.** List of acronyms used in the paper.

AOTF	acousto-optical tunable filter
CFA	color filter array
CS	compressive sensing
FPGA	field programmable gate array
FS1-2	Filter Spatial Distributions 1 and 2
FWHM	full width at half maximum
LCTF	liquid crystal tunable filter
LMMSE	linear minimum mean square error
MMSE	minimum mean square error
MSFA	multispectral filter array
MSI	multispectral imaging
NIR	near-infrared
NUV	near-ultraviolet
PSI	polarization Sagnac interferometer
PSNR	peak signal-to-noise ratio
SSIM	structural similarity
SWIR	short wave infrared
TFD	transverse field detector

**Table 2.** Optical specifications of filter bands, theoretical simulation (Sim.) and practical result.

**Band**	**Central Wave-Length (nm)**		**FWHM (nm)**		**Max Trans-Mission *T****_max_* **(%)**		**Effective Band at 5% × *T****_max_* **(nm)**	

	**Sim.**	**Result**	**Sim.**	**Result**	**Sim.**	**Result**	**Sim.**	**Result**
P1	420	427	35	38	70	47	*NUV* – 473	*NUV* – 495
P2	465	467	26	31	64	50	*NUV* –512	*NUV* – 527
P3	515	510	23	28	61	52	425–550	423–567
P4	560	561	21	26	56	54	502–605	488–614
P5	609	605	19	25	57	49	559–644	540–645
P6	645	654	18	24	62	47	611–689	595–702
P7	700	699	15	22	69	45	664–736	645–743
IR	> 865	> 885	-	-	> 75	> 75	> 826	> 847

**Table 3.** Relative normalized values of the sensor response (*ρ_p_*) by the filter, for a given input illuminant and a perfect diffuser.

**Illuminant**	**E**	**D65**	**A**
*R_Sinarback_*	0.47	0.41	0.68
*G_Sinarback_*	1	1	1
*B_Sinarback_*	0.82	0.85	0.48
*P*_1_	0.78	0.78	0.25
*P*_2_	0.94	1	0.41
*P*_3_	0.97	0.91	0.58
*P*_4_	1	0.81	0.80
*P*_5_	0.95	0.67	0.90
*P*_6_	0.92	0.56	1
*P*_7_	0.84	0.45	0.99
*IR*1 (400–780 nm)	0.84	0.60	0.71
*IR*2 (780–1100 nm)	2.84	x	x

**Table 4.** Spectral interference coefficients of the filter bands. The data used is from the practical spectral characteristics of the filters from Figure 10c.

**i,j**	**P1**	**P2**	**P3**	**P4**	**P5**	**P6**	**P7**
P1	1	0.39	0.18	0.06	0.05	0.04	0.04
P2	0.41	1	0.35	0.12	0.05	0.04	0.05
P3	0.19	0.33	1	0.25	0.10	0.05	0.04
P4	0.11	0.15	0.27	1	0.58	0.10	0.05
P5	0.10	0.10	0.13	0.26	1	0.23	0.08
P6	0.11	0.12	0.09	0.09	0.20	1	0.23
P7	0.12	0.12	0.08	0.06	0.08	0.21	1
